# The Effect of Diabetes Mellitus Severity on Foot & Ankle Burn Recovery

**DOI:** 10.3390/ebj5040035

**Published:** 2024-11-08

**Authors:** Sheldon A. McCown, Elliot T. Walters, Alen Palackic, Camila Franco-Mesa, Ashton R. Davis, Phillip H. Keys, Juquan Song, Steven E. Wolf

**Affiliations:** 1 School of Medicine, University of Texas Medical Branch, Galveston, TX 77555, USA; samccown@utmb.edu (S.A.M.); ashtdavi@utmb.edu (A.R.D.); phkeys@utmb.edu (P.H.K.); 2 Department of Surgery, University of Texas Medical Branch, Galveston, TX 77555, USAalen.palackic@bgu-ludwigshafen.de (A.P.); camfranc@utmb.edu (C.F.-M.); 3 Microsurgery, and Burn Center, Department of Hand, Plastic and Reconstructive Surgery, BG Trauma Center, University of Heidelberg, 67071 Ludwigshafen, Germany

**Keywords:** retrospective study, hemoglobin A1c, glycemic control, amputations

## Abstract

Background: Diabetic patients often present with complex limb pathology, resulting in impaired sensation in the distal extremities making tactile injuries such as burns difficult to notice. We posit that poorly controlled diabetes mellitus, evidenced by increasing elevations in hemoglobin A1c, is associated with delayed wound healing and increased complications in burn patients. Methods: The TriNetX Network, a database of 89 million patients across the U.S., was queried for diabetic patients with foot and ankle burns. Patients were divided into four groups based on A1c: properly controlled (<7%), moderately controlled (7–9%), poorly controlled (>9%), and propensity-matched non-diabetic controls. Evaluated outcomes included split-thickness skin grafting, infections, amputations, acute kidney failure (AKF), and mortality within one month of the burn. Results: When comparing the poorly controlled A1c cohort with the properly controlled and moderately controlled A1c cohorts, we found a significant increase in amputations (*p* = 0.042) and cutaneous infections (*p* = 0.0438), respectively. When evaluating non-diabetics to diabetic patients, significantly increased rates of amputations (*p* < 0.0001), cutaneous infections (*p* = 0.0485), systemic infections (*p* = 0.0066), and AKF (*p* = 0.0005) were noted in the latter. Conclusions: Poorly controlled diabetes shows a significant correlation with increased complications following foot and ankle burns, including amputations, infections, and AKF.

## 1. Introduction

Diabetes mellitus is a chronic disease that affects a large portion of the population with type II diabetes affecting an estimated 462 million people worldwide [1,2]. In the United States in particular, 11.3% of the population is affected which translates to a massive disease burden [3]. Furthermore, these patients commonly have complex medical issues including peripheral neuropathy and peripheral vascular disease. Such complications lead to a greater risk of lower extremity injury and delayed wound healing [4]. While the disease itself is associated with major multi-organ complications, those with higher hemoglobin A1c levels also display worsening wound healing due to a disruption in angiogenesis [5]. As a result, complications like infection, extended length of hospital stay, amputation, and increased mortality often arise from injuries such as burns [6,7]. The rising rates of diabetes both globally and in the United States coupled with the associated risks and complications in this population demonstrate the importance of understanding the effects of poorly controlled diabetes in recovery following burn injury specifically. The aim of this study is to examine the effect of poorly controlled diabetes mellitus, evidenced by increasing elevations in hemoglobin A1c, and its associated complications in burn patients. Improved understanding should lead to cost-effective treatments and prevention strategies resulting in improved patient care by healthcare providers. 

## 2. Methods

The data used in this study was collected on 24 December 2022, from the TriNetX Network. TriNetX currently provides access to the electronic medical records of 89 million patients from 59 healthcare organizations (HCO) across the globe. TriNetX is certified to the ISO 27001:2013 standard and maintains an Information Security Management System (ISMS) to ensure the protection of healthcare data and meet the requirements of the HIPAA Security Rule. Patient-level data provided in a data set generated by TriNetX Platform only contains de-identified data as proscribed by standards defined in Section §164.514(a) of the HIPAA Privacy Rule. Institutional Review Board approval was given prior to the study (UTMB IRB # 20-0085).

TriNetX was used to evaluate the effect of diabetes mellitus stratified by A1c levels on outcomes following foot and ankle burns. Cohorts were divided into three groups: properly controlled (Alc: <7%), moderately controlled (A1c: 7–9%), and poorly controlled (A1c: >9%). To qualify for these cohorts, patients required a prior diagnosis of diabetes mellitus (ICD-10-CM: E08-E13) and a burn injury of the foot or ankle (ICD-10-CM: T25). Patients with burns on more than one body site in addition to a foot or ankle burn were excluded. Next, patients were divided into three cohorts based on their most recent A1c level. The A1c level was within one year of burn injury. After establishing the three cohorts, three separate comparisons were created: properly controlled vs. moderately controlled, moderately controlled vs. poorly controlled, and properly controlled vs. poorly controlled. 

We made a fourth comparison of outcomes in those with moderately (A1c: 7–9%) or poorly controlled (A1c: >9%) diabetes to those without diabetes (A1c: <6.5%). To qualify for the diabetes cohort, a diagnosis of diabetes mellitus (ICD-10-CM: E08-E13) and a foot or ankle burn (ICD-10-CM: T25) occurring after the most recent A1c > 7 was required, combining the moderately controlled A1c and poorly controlled A1c cohorts. Well-controlled diabetes is <7%. Moderate is between 7 and 9% and poorly controlled is 9% or above. The reference is from Electronic Clinical Quality Improvement (eCQI) Resource Center (CMS122v12). https://ecqi.healthit.gov/ecqm/ec/2024/cms0122v12?qt-tabs_measure=measure-information (Accessed on 1 August 2024). For the non-diabetes cohort, diabetes mellitus was used as an exclusion criterion, and the patient required a foot or ankle burn after the most recent A1c: <6.5, the A1c required for the diagnosis of diabetes.

**Figure 1:**
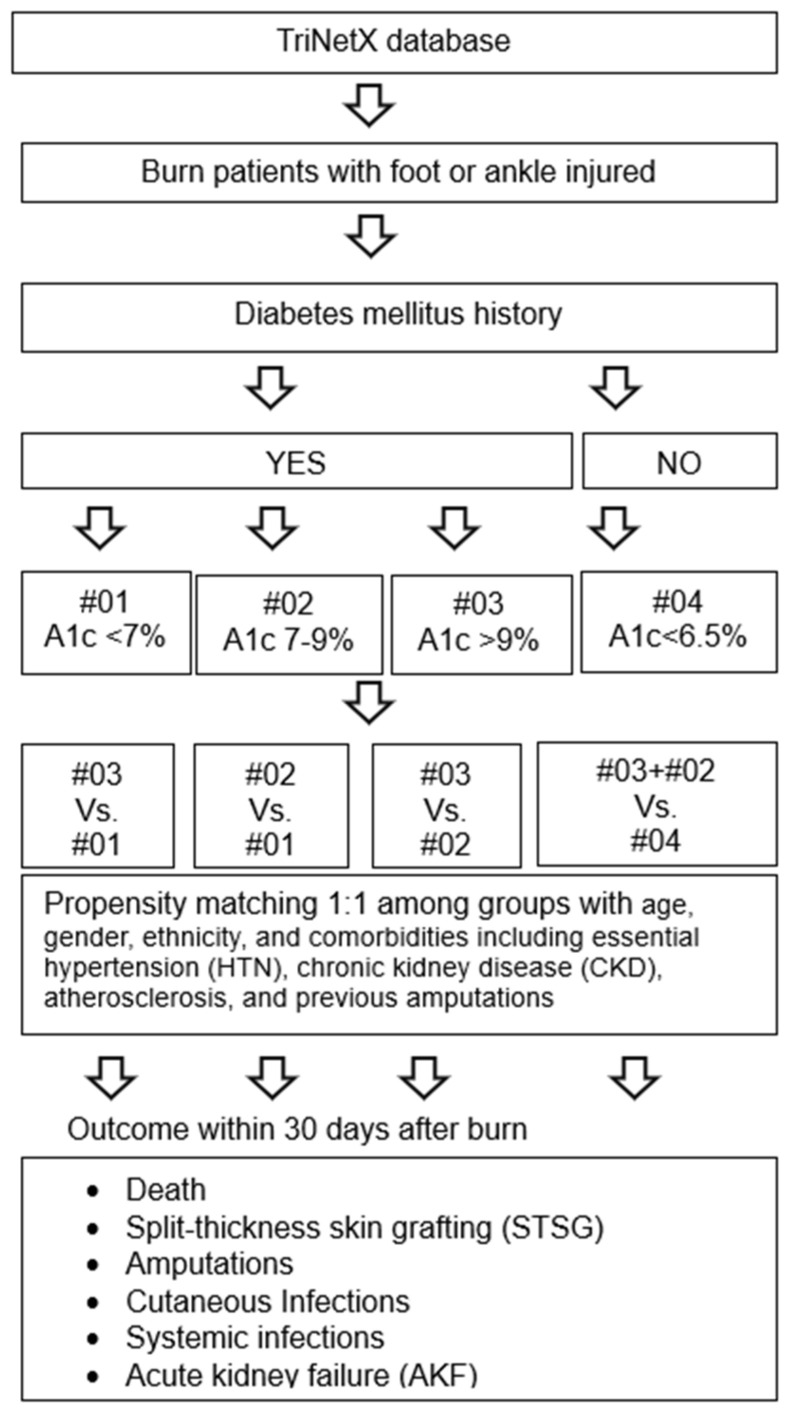
The flow chart of the study design. Four subgroups (#01–04) were formatted by A1c levels in patients, and four comparisons were formed based on four subgroups. The principle analysis was conducted on 24 December 2022 with additional data being pulled a few days later. Patients were not removed from the study and all analyses went through the same matching process.

For each comparison, cohorts were propensity-score matched by age, gender, ethnicity, and comorbidities including essential hypertension (HTN) (ICD-10-CM: I10), chronic kidney disease (CKD) (ICD-10-CM: N18), atherosclerosis (ICD-10-CM: I25.10), and previous amputations (ICD-10-CM: Z89). The primary outcomes of the study were evaluated from the time of burn through 30 days following the injury. Outcomes evaluated include univariate statistical analysis of outcomes performed using the analytical tools provided by the TriNetX database, which included paired t-tests, along with measures of association including odds ratios with a 95% confidence interval. A *p*-value less than 0.05 was considered significant (Figure 1). 

## 3. Results

Prior to matching, the properly controlled diabetes cohort (A1c < 7%) had 1079 patients, 58.08% male, and 41.92% female, with an average age of 59.3 ± 15.3. The moderately controlled diabetes cohort (A1c 7–9%) had 675 patients, 68% male, and 29.48% female, with an average age of 60.9 ± 13.1. The poorly controlled diabetes cohort A1c (>9%) had 591 patients prior to matching, 67.85% male and 30.46% female, with an average age of 53.7 ± 13. The no diabetes cohort (A1c < 6.5%) had 2151 patients, 67.96% male, and 30.03% female, with an average age of 48.8 ± 19.2. Full demographic data can be found in Table 1, Table 2, Table 3 and Table 4.

Since four separate groups were included, cohorts were analyzed using multiple paired t-tests. Prior to matching, all four comparison setups had significant differences in age, gender, ethnicity, and comorbidities; however, significant differences in nearly all categories were eliminated after matching.

Comparing the properly controlled A1c: (Alc: <7%) and poorly controlled Alc (A1c: >9%) groups, we found a significant risk of foot and toe amputations in the latter (*p* = 0.042, OR: 1.69, 95% CI: [1.014, 2.826]). However, no significant difference was seen regarding mortality (*p* = 0.8671, OR: 0.95, 95% CI: [0.491, 1.822]), grafting rates (*p* = 0.7288, OR: 1.08, 95% CI: [0.689, 1.705]), cutaneous infections (*p* = 0.1052, OR: 1.56, 95% CI: [0.908, 2.675]), systemic infections (*p* = 0.2308, OR: 1.27, 95% CI: [0.858, 1.884]), or AKF (*p* = 0.1554, OR: 1.34, 95% CI: [0.895, 1.995]) (Table 5).

Regarding the properly controlled A1c (Alc: <7%) and moderately controlled A1c (Alc: 7–9%) comparisons, we found a significant risk of cutaneous infections in the properly controlled A1c group (*p* = 0.0421, OR: 0.57, 95% CI: [0.328, 0.986]). However, we found no significant differences in mortality (*p* = 0.2093, OR: 0.67, 95% CI: [0.359, 1.255]), grafting rates (*p* = 0.3379, OR: 1.26, 95% CI: [0.785, 2.019]), foot or toe amputations (*p* = 0.7881, OR: 1.08, 95% CI: [0.635, 1.821]), systemic infections (*p* = 0.5786, OR: 0.90, 95% CI: [0.672, 1.297]), or AKF (*p* = 0.2153, OR: 0.80, 95% CI: [0.566, 1.137]) (Table 6).

Between the moderately controlled A1c (A1c: 7–9%) and poorly controlled A1c (A1c: >9%) groups, we found a significant risk of cutaneous infections in the poorly controlled A1c group (*p* = 0.0438, OR: 1.74, 95% CI: [1.01, 2.995]). No significant difference was seen regarding mortality (*p* = 0.2498, OR: 1.57, 95% CI: [0.725, 3.378]), grafting rates (*p* = 0.4835, OR: 0.85, 95% CI: [0.536, 1.343]), foot or toe amputations (*p* = 0.1755, OR: 1.45, 95% CI: [0.845, 2.483]), systemic infections (*p* = 0.6081, OR: 1.11, 95% CI: [0.743, 1.661]), or AKF (*p* = 0.4248, OR: 1.17, 95% CI: [0.793, 1.736]) (Table 7).

When comparing patients with moderately or poorly controlled diabetes and those without, the diabetes group showed a significant increase in rates of foot and toe amputations (*p* <0.0001, OR: 6.07, 95% CI: [3.084, 11.93]), cutaneous infections (*p* = 0.0485, OR: 1.51, 95% CI: [1, 2.292]), systemic infections (*p* = 0.0066, OR: 1.53, 95% CI: [1.124, 2.087]), and AKF (*p* = 0.0005, OR: 1.72, 95% CI: [1.268, 2.345]). Nonetheless, no significant difference was seen in mortality (*p* = 0.302, OR: 1.36, 95% CI: [0.757, 2.437]) or grafting rates (*p* = 0.1085, OR: 1.28, 95% CI: [0.946, 1.729]) (Table 8).

## 4. Discussion

Based on these findings, patients with poorly controlled diabetes have a greater likelihood of complications within one month following burn injury than their properly controlled counterparts. Specifically, patients with poorly controlled diabetes are significantly more likely to receive foot and toe amputations and develop cutaneous infections. Additionally, poorly controlled diabetic patients are significantly more likely to undergo foot or toe amputations as well as develop systemic infections, cutaneous infections, and acute kidney failure compared to properly controlled and non-diabetic counterparts. Rates of split-thickness skin grafting (STSG) varied among the different cohorts. The study itself analyzes burns in general, not the effect of burn depth and size. Severely elevated A1c was shown to be correlated with greater complications following lower extremity burns, the degree of diabetes appears to be a more significant risk factor for complications following burn injury.

Despite the high prevalence of burn injuries in diabetic populations, we found only a few studies regarding how disease severity affects the wound healing process. Shalom et al. reported that diabetic patients underwent operative wound closure in a noticeably higher proportion than non-diabetics, 72.6% vs. 32% (*p* < 0.01), despite comparable total body surface area (TBSA) burn values [8]. Furthermore, higher mortality and longer hospitalization stays in the diabetic cohort were also seen [8]. Similarly, McCampbell et al. noted that while TBSA burn size was comparable between diabetics and non-diabetics, the diabetic cohort had higher rates of infection (64.9% vs. 50.5%, *p* = 0.05) and full-thickness burns (51% vs. 32%, *p* = 0.025); however, mortality rates were similar between the two groups (2.1% vs. 2.2%) [9]. STGS rates varied across cohorts in our study but were found to be higher in diabetics likely due to more severe burns requiring skin grafting. Another study described that diabetics had significantly longer median length of hospital stay per TBSA burn size (2.1 vs. 1.6 days, *p* = 0.0026) and a greater overall morbidity (1.39 +/− 1.63 vs. 0.8 +/− 1.24; *p* = 0.001) than the non-diabetic counterparts [7]. As Born et al. depicted in their meta-analysis, diabetics have 2.38 times higher odds of mortality, 5.47 times higher odds of wound and soft tissue infections, and 37 times higher odds of undergoing amputation compared to non-diabetic patients after suffering a burn injury [10]. While Born et al. also identified the significantly elevated rates of AKI in the diabetic population after burn injury, their research was further completed by noticing increased respiratory failure and heart failure in this population. Alternatively, a study conducted by Dahagam et al. evaluated clinical outcomes in patients with a previous history of diabetes and found no significant association between a pre-existing diagnosis of diabetes and negative clinical outcomes [11]. However, this study did find that these patients exhibited more extreme physiologic and metabolic derangements when compared to their non-diabetic counterparts, requiring more intensive glucose control during hospitalization. The aforementioned studies point to complications in diabetic burn injuries including higher mortality, higher rates of infection, increased amputation rates, greater AKI occurrence, increased length of stay, and significant metabolic abnormalities. Our study supports these findings as we found significantly increased rates of infections, amputations, and AKF among diabetic patients. Our study found no difference in mortality, which is likely related to the small TBSA of foot and ankle burns which are often non-life threatening. If serious infection or injury occurs to the foot, amputation is likely to proceed before death becomes a serious concern. The previous studies included all burn injuries, rather than specifically evaluating foot and ankle burns as we did in our study, which is a crucial distinction as diabetic patients often have complex pathologies of the lower extremity. It was also found that properly controlled diabetics compared to poorly controlled diabetics had a higher risk of cutaneous infections. The remaining comparison groups consistently demonstrated a higher percentage of cutaneous infections in those with higher A1c values. The authors presume that the majority, if not all of the cutaneous infections are at the site of the burn. However, it is possible that a patient incidentally has cellulitis or another local skin infection that was included in their diagnosis at the time of the burn. All non-local infections were calculated as systemic infections per ICD-10 coding. We did not include sepsis as that is a different ICD-10 code

When specifically considering foot and ankle burns, diabetic patients were 1.7 times more likely to sustain an injury in winter than non-diabetics according to Goutos et al. [12]. This population was also 3.8 times more likely to have contact burns compared to the healthy control group (*p* < 0.001) [13]. Not only do diabetic patients have greater comorbidities associated with burn injuries, but they are also more likely to sustain the burn injury itself. An explanation for this phenomenon likely lies in the impact that diabetic neuropathy has on sensation and proprioception of the lower extremities. Diabetic patients with advanced neuropathy have decreased sensation to pain and heat, which predisposes them to more severe burn injuries as they may not even notice the burn injury as it occurs. On top of poor sensation, many diabetics also suffer from poor circulation in the lower extremity leading to decreased blood supply and dampened healing ability. Paired with the findings from our study, current literature shows that diabetics not only have greater rates of burn injury on the lower extremities but also have greater complications when those burn injuries occur.

Regarding the outcomes associated with the severity of disease based on A1c levels, Murphy et al. found that elevated A1c results in higher rates of unplanned readmission rates for burns (18.8% vs. 3.6%, *p* = 0.001) and a longer length of stay for burn care (13 days vs. 9 days, *p* = 0.038). Despite this, no difference in mortality was encountered [14]. Dolp et al. found similar results as patients with an A1c > 7% had an increased median length of stay per TBSA burned (2.1 vs. 1.6 days, *p* = 0.0026) and greater overall morbidity (1.39 vs. 1.24, *p* = 0.001) [7]. Reinforcing our results, these articles reported that increasing severity of diabetes (evidenced by higher levels of A1c) is associated with greater severity of complications following burns. While these studies support our results by showing that elevated A1c is associated with greater severity of complications following burns, they lack a stepwise stratification of diabetes severity based on the A1c that our study provides. By dividing diabetes based on the severity of A1c elevation, we are able to demonstrate that not only the presence of diabetes but the severity of diabetes is associated with more severe complications following lower extremity burns.

Our findings point to increased complications following foot and ankle burns in patients with poorly controlled diabetes, which emphasizes the need for appropriate glycemic control in the pre-injury setting. However, pre-injury glycemic control is not modifiable at the time of burn presentation, so actionable changes in patient care should be taken to treat these patients and attempt to counter the negative impact of chronically elevated glucose. The first step is recognizing the severity of diabetes with A1c levels drawn at the time of assessment. This practice enables providers to predict complications regarding inpatient glucose control and facilitates an attempt to prevent negative clinical outcomes associated with hyperglycemia [11]. Following confirmation of diabetes severity, several postulated treatment options exist to decrease complications in these patients, one of which is the use of growth factors which have previously been used to treat nonhealing diabetic ulcers [15]. As diabetics lack sufficient growth factors, supplementation of these factors may increase wound healing, which in turn decreases rates of infection and amputation [16,17]. Furthermore, overtreatment of hyperglycemia in the ICU setting should be avoided. Finfer et al. and the NICE-SUGAR investigators showed that intensive glucose control increases mortality among adults in the ICU, and a glucose target of 180 mg or less per deciliter is an appropriate treatment goal [18]. Lastly, all diabetic patients should be educated on the hazards associated with burn injuries. As they often have impaired temperature sensation, proprioception, and reflexes, they should be educated on taking proactive actions such as checking water temperature with a thermometer rather than relying on their often insensate feet to limit the likelihood of developing a lower extremity burn [19]. Diabetes is a disease that currently has no cure; however, steps such as the ones previously mentioned may help decrease the risk of severe complications in diabetic patients following burns.

Besides the inherent limitations known to retrospective scientific studies, drawbacks associated with the administrative data collection were encountered in this project. Specifically, there is a marked dependence on accuracy regarding coding diagnosis, lab values, and treatments to yield an appropriate chronological search. Given the de-identified character of the database, certain in-depth details such as accurate descriptions of the burn wounds with measurements and specific locations within the feet are unavailable for evaluation, limiting the span of the analysis. Burn depth was not included in the study and thus is a limitation to understanding rates of STSG. To decrease the limitations and bias encountered, all the patients are matched for confounding variables and comorbidities. 

## 5. Conclusions

As in other reports in the literature, our findings suggest that poorly controlled diabetes evidenced by an elevated A1c is associated with increased complications following foot and ankle burn injury when compared to non-diabetics and those with properly controlled diabetes, specifically regarding infection and amputation rates. These results emphasize the importance of proper glycemic control in the pre-injury setting and prompt the need for intensive and coordinated care after injury for poorly controlled diabetic burn patients.

## Figures and Tables

**Table 1:** Demographics (A1c: >9% vs. A1c: <7%).

	Prior to Propensity Match					Post Propensity Match				
	A1c: >9%		A1c: <7%		*p*-Value	A1c: >9%		A1c: <7%		*p*-Value
Total Patients	591		1071			536		536		
Age	53.7 ± 13		59.3 ± 15.3		<0.0001 *	54.4 ± 12.8		54.6 ± 15		0.8991
Gender										
Male	401	67.85%	622	58.08%	<0.0001 *	360	67.16%	358	66.79%	0.8967
Female	180	30.46%	449	41.92%	<0.0001 *	175	32.65%	177	33.02%	0.8965
Ethnicity										
White	323	54.65%	717	66.95%	<0.0001 *	306	57.09%	300	55.97%	0.7116
Black or African American	158	26.73%	204	19.05%	0.0001 *	138	25.75%	144	26.87%	0.6773
Hispanic/Latino	90	15.23%	86	8.03%	<0.0001 *	69	12.87%	71	13.25%	0.8561
Not Hispanic/Latino	408	69.04%	859	80.21%	<0.0001 *	390	72.76%	390	72.76%	1
Unknown Ethnicity	84	14.21%	129	12.04%	0.1599	77	14.37%	75	13.99%	0.861
Unknown Race *	84	14.21%	114	10.64%	0.0222 *	75	13.99%	73	13.62%	1
Comorbidities										
Essential HTN	325	54.99%	771	71.99%	<0.0001 *	313	58.40%	327	61.01%	0.3833
Chronic Kidney Disease	113	19.12%	324	30.25%	<0.0001 *	110	20.52%	101	18.84%	0.4893
Atherosclerosis	110	18.61%	278	25.96%	0.0014 *	108	20.15%	106	19.78%	0.8785
Acquired Absence of Limb (Amputations)	56	9.48%	87	8.12%	0.2926	54	10.07%	35	6.53%	0.0354 *

* a group of people sharing a common cultural, geographical, linguistic, or religious origin or background.

**Table 2:** Demographics (A1c: 7–9% vs. A1c: <7%).

	Prior to Propensity Match					Post Propensity Match				
	A1c: 7–9%		A1c: <7%		*p*-Value	A1c: 7–9%		A1c: <7%		*p*-Value
Total Patients	675		1079			635		635		
Age	60.9 ± 13.1		59.3 ± 15.3		0.0257 *	60.7 ± 13.2		61 ± 13.8		0.7332
Gender										
Male	459	68.00%	622	57.65%	<0.0001 *	433	68.19%	427	67.24%	0.7188
Female	199	29.48%	449	41.61%	<0.0001 *	199	31.34%	205	32.28%	0.7177
Ethnicity										
White	444	65.78%	717	66.45%	0.8597	425	66.93%	428	67.40%	0.8577
Black or African American	126	18.67%	204	18.91%	0.9722	122	19.21%	119	18.74%	0.83
Hispanic/Latino	47	6.96%	86	7.97%	0.4952	46	7.24%	44	6.93%	0.8269
Not Hispanic/Latino	507	75.11%	859	79.61%	0.1050	494	77.80%	493	77.64%	0.9462
Unknown Ethnicity	107	15.85%	129	11.96%	0.0137 *	95	14.96%	98	15.43%	0.8146
Unknown Race *	69	10.22%	114	10.57%	0.9078	66	10.39%	67	10.55%	1
Comorbidities										
Essential HTN	435	64.44%	771	71.46%	0.0086 *	428	67.40%	427	67.24%	0.9523
Chronic Kidney Disease	194	28.74%	324	30.03%	0.7176	189	29.76%	184	28.98%	0.758
Atherosclerosis	188	27.85%	278	25.76%	0.2432	178	28.03%	178	28.03%	1
Acquired Absence of Limb (Amputations)	50	7.41%	87	8.06%	0.6875	49	7.72%	53	8.35%	0.6796

* a group of people sharing a common cultural, geographical, linguistic, or religious origin or background.

**Table 3:** Demographics (A1c: >9% vs. A1c: 7–9%).

	Prior to Propensity Match					Post Propensity Match				
	A1c: >9%		A1c: 7–9%		*p*-Value	A1c: >9%		A1c: 7–9%		*p*-Value
Total Patients	627		724			480		480		
Age	53.7 ± 12.9		61.1 ± 13.1		<0.0001 *	56.7 ± 12		56.6 ± 12.1		0.8997
Gender										
Male	429	68.42%	489	67.54%	0.8304	330	68.75%	337	70.21%	0.6237
Female	188	29.98%	218	30.11%	0.9109	149	31.04%	142	29.58%	0.623
Ethnicity		0.00%		0.00%			0.00%		0.00%	
White	343	54.70%	474	65.47%	<0.0001 *	288	60.00%	292	60.83%	0.7918
Black or African American	161	25.68%	128	17.68%	0.0004 *	113	23.54%	109	22.71%	0.7595
Hispanic/Latino	107	17.07%	59	8.15%	<0.0001 *	62	12.92%	57	11.88%	0.6243
Not Hispanic/Latino	427	68.10%	542	74.86%	0.003 *	349	72.71%	357	74.38%	0.5582
Unknown Ethnicity	84	13.40%	109	15.06%	0.3641	69	14.38%	66	13.75%	0.7806
Unknown Race *	97	15.47%	86	11.88%	0.5880	66	13.75%	65	13.54%	1
Comorbidities										
Essential HTN	342	54.55%	463	63.95%	0.0002 *	284	59.17%	282	58.75%	0.8956
Chronic Kidney Disease	120	19.14%	204	28.18%	<0.0001 *	106	22.08%	96	20.00%	0.4285
Atherosclerosis	113	18.02%	199	27.49%	<0.0001 *	105	21.88%	97	20.21%	0.5264
Acquired Absence of Limb (Amputations)	59	9.41%	54	7.46%	0.206	46	9.58%	33	6.88%	0.1268

* a group of people sharing a common cultural, geographical, linguistic, or religious origin or background.

**Table 4:** Demographics (Diabetes vs. No Diabetes).

	Prior to Propensity Match					Post Propensity Match				
	Diabetes **		No Diabetes **		*p*-Value	Diabetes **		No Diabetes **		*p*-Value
Total Patients	1342		2151			1107		1107		
Age	57.6 ± 13.5		48.8 ± 19.2		<0.0001 *	55.9 ± 13.2		58.2 ± 15.5		0.0002 *
Gender										
Male	912	67.96%	1178	54.77%	<0.0001 *	732	66.12%	718	64.86%	0.5314
Female	403	30.03%	945	43.93%	<0.0001 *	371	33.51%	387	34.96%	0.4736
Ethnicity										
White	809	60.28%	1379	64.11%	0.0607	681	61.52%	715	64.59%	0.1344
Black or African American	289	21.54%	431	20.04%	0.2180	244	22.04%	245	22.13%	0.9591
Hispanic/Latino	165	12.30%	227	10.55%	0.0883	135	12.20%	113	10.21%	0.1382
Not Hispanic/Latino	962	71.68%	1620	75.31%	0.0623	819	73.98%	853	77.06%	0.0929
Unknown Ethnicity	192	14.31%	291	13.53%	0.4360	153	13.82%	141	12.74%	0.4523
Unknown Race *	182	13.56%	258	11.99%	0.1380	148	13.37%	114	10.30%	0.0253 *
Comorbidities										
Essential HTN	792	59.02%	771	35.84%	<0.0001 *	600	54.20%	619	55.92%	0.4169
Chronic Kidney Disease	314	23.40%	147	6.83%	<0.0001 *	168	15.18%	145	13.10%	0.1606
Atherosclerosis	306	23.20%	162	7.58%	<0.0001 *	180	16.26%	155	14.00%	0.1382
Acquired Absence of Limb (Amputations)	109	8.12%	22	1.02%	<0.0001 *	83	7.50%	15	1.36%	<0.0001*

* a group of people sharing a common cultural, geographical, linguistic, or religious origin or background. ** Diabetes group = moderately (A1c: 7–9%) and poorly controlled (A1c: >9%); No Diabetes = A1c: <6.5%.

**Table 5:** Outcomes (A1c: >9% vs. A1c: <7%): Outcomes between the poorly controlled and properly controlled cohorts were evaluated within the first month following the burn injury; STSG = split-thickness skin grafting; AKF = acute kidney failure.

Outcome	A1c: >9%		A1c: <7%		*p*-Value	Odds Ratio	Odds Cl
Death	18	3.36%	19	3.54%	0.8671	0.95	(0.491, 1.822)
STSG	42	7.84%	39	7.28%	0.7288	1.08	(0.689, 1.705)
Amputations	41	7.65%	25	4.66%	0.042 *	1.69	(1.014, 2.826)
Cutaneous Infections	35	6.53%	23	4.29%	0.1052	1.56	(0.908, 2.675)
Systemic Infections	62	11.57%	50	9.33%	0.2308	1.27	(0.858, 1.884)
AKF	61	11.38%	47	8.77%	0.1554	1.34	(0.895, 1.995)

*, *p* < 0.05 with statistical significance.

**Table 6:** Outcomes (A1c: 7–9% vs. A1c: <7%): Outcomes between the moderately controlled and properly controlled cohorts were evaluated within the first month following the burn injury; STSG = split-thickness skin grafting; AKF = acute kidney failure.

Outcome	A1c: 7–9%		A1c: <7%		*p*-Value	Odds Ratio	Odds Cl
Death	17	2.68%	25	3 .94%	0.2093	0.67	(0.359, 1.255)
STSG	41	6.46%	33	5.20%	0.3379	1.26	(0.785, 2.019
Amputations	30	4.72%	28	4.41%	0.7881	1.08	(0.635, 1.821)
Cutaneous Infections	21	3.31%	36	5.67%	0.0421 *	0.57	(0.328, 0.986)
Systemic Infections	62	9.76%	68	10.71%	0.5786	0.90	(0.672, 1.297)
AKF	65	10.24%	79	12.44%	0.2153	0.80	(0.566, 1.137)

*, *p* < 0.05 with statistical significance.

**Table 7:** Outcomes (A1c: >9% vs. A1c: 7–9%): Outcomes between the poorly controlled and moderately controlled cohorts were evaluated within the first month following the burn injury; STSG = split-thickness skin grafting; AKF = acute kidney failure.

Outcome	A1c: >9%		A1c: 7–9%		*p*-Value	Odds Ratio	Odds Cl
Death	17	3.54%	11	2.29%	0.2498	1.57	(0.725, 3.378)
STSG	37	7.71%	43	8.96%	0.4835	0.85	(0.536, 1.343)
Amputations	34	7.08%	24	5.00%	0.1755	1.45	(0.845, 2.482)
Cutaneous Infections	37	7.71%	22	4.58%	0.0438 *	1.74	(1.01, 2.995)
Systemic Infections	56	11.67%	51	10.63%	0.6081	1.11	(0.743, 1.661)
AKF	61	12.71%	53	11.04%	0.4248	1.17	(0.793, 1.736)

*, *p* < 0.05 with statistical significance.

**Table 8:** Outcomes (Diabetes [moderately and poorly controlled] vs. No Diabetes [A1c: <6.5%]): Outcomes between the combined diabetic cohort and the propensity-matched non-diabetic cohort were evaluated within the first month following the burn injury; STSG = split-thickness skin grafting; AKF = acute kidney failure.

Outcome	Diabetes		No Diabetes		*p*-Value	Odds Ratio	Odds Cl
Death	27	2.44%	20	1.81%	0.302	1.36	(0.757, 2.437)
STSG	104	9.39%	83	7.50%	0.1085	1.28	(0.946, 1.729)
Amputations	58	5.24%	10	0.90%	<0.0001 *	6.07	(3.084, 11.93)
Cutaneous Infections	58	5.24%	39	3.52%	0.0485 *	1.51	(1, 2.292)
Systemic Infections	108	9.76%	73	6.59%	0.0066 *	1.53	(1.124, 2.087)
AKF	117	10.57%	71	6.41%	0.0005 *	1.72	(1.268, 2.345 )

*, *p* < 0.05 with statistical significance.

## Data Availability

All relevant data are available within the presented manuscript. Any material and information generated during the study will be available for sharing with other researchers under appropriate institutional agreements. Any inquiries should be directed to corresponding authors Drs. Song and Wolf.
